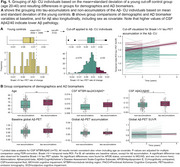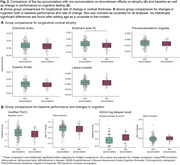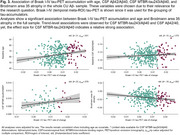# Longitudinal tau‐PET accumulation and downstream effects in amyloid‐beta negative cognitively unimpaired individuals

**DOI:** 10.1002/alz70856_104826

**Published:** 2026-01-07

**Authors:** Anika Wuestefeld, Nicola Spotorno, Alexa Pichet Binette, Niklas Mattsson‐Carlgren, Rik Ossenkoppele, Sebastian Palmqvist, Ruben Smith, Erik Stomrud, Olof Strandberg, Danielle van Westen, Oskar Hansson, Laura E.M. Wisse

**Affiliations:** ^1^ Clinical Memory Research Unit, Department of Clinical Sciences Malmö, Lund University, Lund, Sweden; ^2^ Clinical Memory Research Unit, Department of Clinical Sciences Malmö, Lund University, MOntreal, QC, Canada; ^3^ Department of Physiology and Pharmacology, Université de Montréal, Montréal, QC, Canada; ^4^ Centre de recherche de l'institut universitaire de gériatrie de Montréal (CRIUGM), Montréal, QC, Canada; ^5^ Clinical Memory Research Unit, Lund University, Malmö, Skåne, Sweden; ^6^ Memory Clinic, Skåne University Hospital, Malmö, Skåne, Sweden; ^7^ Wallenberg Center for Molecular Medicine, Lund University, Lund, Sweden; ^8^ Clinical Memory Research Unit, Department of Clinical Sciences Malmö, Faculty of Medicine, Lund University, Lund, Sweden; ^9^ Imaging and Function, Skåne University Hospital, Lund, Sweden; ^10^ Department of Diagnostic Radiology, Clinical Sciences, Lund University, Lund, Sweden; ^11^ Diagnostic Radiology, Department of Clinical Sciences Lund, Lund University, Lund, Sweden

## Abstract

**Background:**

Previous studies, including ours (Wuestefeld et al., *Brain*, 2023), showed tau positron emission tomography (PET) uptake in medial temporal and neocortical regions of amyloid‐beta (Aβ)‐negative cognitively unimpaired (CU) individuals, associated with neurodegeneration and worse memory performance. To further understand if tau‐PET uptake is clinically relevant in this population, we characterized Aβ‐negative CU individuals with higher longitudinal tau‐PET accumulation and its associations with atrophy and cognitive decline.

**Method:**

We included 333 CU BioFINDER‐2 participants, negative for both global [18F]flutemetamol Aβ‐PET and cerebrospinal fluid (CSF) Aβ42/Aβ40 (age=63.8, 55% female, 2.28±1.36 years follow‐up). Using linear mixed‐effects models, we calculated the rate of change (ROC) in [18F]RO948 tau‐PET SUVR in composite regions of interest (ROI), recapitulating the Braak stages. Individuals were classified as “tau‐accumulators” vs. “non‐accumulators” based on the mean+standard deviation of Braak I‐IV tau‐PET ROC in young controls (20‐40 years, *n* = 29; Figure 1A). Tau‐PET ROC and thickness of the entorhinal cortex, Brodmann area (BA)35 and neocortical AD‐regions (precuneus/posterior cingulate, lateral occipital, superior frontal) were extracted. Group differences and associations with CSF AD biomarkers, delayed word‐list recall, and modified Preclinical Alzheimer Cognitive Composite were tested.

**Result:**

16% of individuals were identified as tau‐accumulators. They were older, had lower levels of Aβ as indicated by a higher CSF Aβ42/40 ratio (no difference on Aβ‐PET) and numerically higher CSF MTBR‐tau243/Aβ40 (not significant, note missingness; Figure 1B+C). Tau‐accumulators showed significantly greater tau‐PET at baseline and accumulation in regions not included in Braak I‐IV (all *p* <0.001). Additionally, we observed more atrophy in BA35 (Figure 2A, not after age‐adjustment) and numerically worse baseline cognitive performance, but no difference in decline over time (Figure 2B).

In the whole sample, Braak I‐IV tau‐PET accumulation was significantly associated with increased age, more BA35 atrophy and at trend‐level with higher levels of CSF Aβ42/40 and CSF MTBR‐tau243/Aβ40 (Figure 3).

**Conclusion:**

A subgroup of Aβ‐ CU individuals show longitudinal tau accumulation across the neocortex. Preliminary results suggest an association with a tau‐specific CSF biomarker and focal atrophy, which did not translate to changes in cognition. We will complement our analyses with a data‐driven approach of classifying tau‐accumulators.